# Commencements

**Published:** 1885-08

**Authors:** 


### EDUCATIONAL.

### COMMENCEMENTS.

ON THIS AND THE SUCCEEDING PAGES ARE GIVEN THE NAMES OF THE GRADUATES OF THE VARIOUS DENTAL COLLEGES OF THE UNITED STATES FOR THE TERM OF 1884-85.

### Baltimore College.

The forty-sixth commencement of this Institution was held on Thursday, March 5th.

| NAME. | RESIDENCE. |
|---|---|
| Eugene F. Adair | Georgia. |
| H. Clay Anders | Maryland. |
| John M. Anderson | Virginia. |
| J. A. Breland | South Carolina. |
| G. P. Chaponis | France. |
| Thos. L. Cobb | Alabama. |
| Ola B. Comfort | Pennsylvania. |
| E. E. Early | Maryland. |
| C. H. Gatewood | Virginia. |
| H. H. Hafer | Georgia. |
| J. E. Hancock | North Carolina. |
| M. Parke Harris | California. |
| L. Hedrick | California. |
| Samual H. Jones | New York. |
| J. C. Morgan | Kentucky. |
| Herbert Phillips | Massachusetts. |
| C. G. Richardson | South Carolina. |
| Phineas A. Sherman | Massachusetts. |
| G. Marshall Smith | Maryland. |
| W. B. Sprinkle | Virginia. |
| J. M. Staire | Pennsylvania. |
| Claude A. St. Amand | South Carolina. |
| N. A. Strait | Dist. of Columbia. |
| R. E. Sunderlin | New York. |
| J. H. Swartz | Pennsylvania. |
| Geo. S. Todd | Maryland. |
| R. C. Warfield | Maryland. |
| F. K. White | Maryland. |

Number of Matriculates, seventy-nine. Graduates, twenty-eight.

### Ohio College of Dental Surgery.

The thirty-ninth commencement of the Ohio College of Dental Surgery was held Wednesday, March 4th.

| NAME. | RESIDENCE. |
|---|---|
| G. F. Ambrose | Illinois. |
| John S. Chance | Ohio. |
| Charles Clark | Ohio. |
| Walter L. Conkey | Wisconsin. |
| John W. Cosford | Michigan. |
| Albert Doerler | Ohio. |
| William Robert Edgar | Ohio. |
| Charles P. Gray | Ohio. |
| Harry H. Genslinger | Ohio. |
| Edward R. Hoff | Ohio. |
| C. R. Holt | Oregon. |
| W. P. Jackson | Ohio. |
| Harry M. Kempton | Ohio. |
| Frank L. King | Pennsylvania. |
| Geo. M. Kinsey | Indiana. |
| J. Fred. Kruger | Ohio. |
| Carrie Lloyd | Indiana. |
| Ben. W. McPhee | Colorado. |
| Louis G. Meyer | Ohio. |
| James D. Moore | Ohio. |
| J. E. Morton | Indiana. |
| Adelbert T. Olmstead | Illinois. |
| A. W. Paffenbarger | Ohio. |
| Homer W. Pitner | Illinois. |
| William M. Seeger | Wisconsin. |
| Jerome B. Williams | Wisconsin. |

Number of matriculates, fifty-nine. Graduates, twenty-six.

### Pennsylvania College of Dental Surgeons.

The twenty-ninth annual commencement was held Feb. 26th, when the degree of D.D.S. was conferred on the following persons:

| NAME. | RESIDENCE. |
|---|---|
| I. H. F. Albrecht | Germany. |
| Deogracias Ascencio | U.S. of Col., S.A. |
| Thomas James Barrett | Massachusetts. |
| J. Irwin Bayles | New Jersey. |
| L.N. Bedford | Iowa. |
| Franz Binotsch | Germany. |
| William Blanc. | New Jersey. |
| Librado Borja | Mexico. |
| Carlos Bonilla | Nicaragua, C. A. |
| Johanna von Bremen | Germany. |
| Frederick H. Brown | New Hampshire. |
| Frank P. Cobourn | Pennsylvania. |
| William B. Connor | Ohio. |
| Frank G. Cooper. | Pennsylvania. |
| Alex J. Culbertson | Pennsylvania. |
| Samuel B. Detweiler | Pennsylvania. |
| Carlton L. Dobbins | New Jersey. |
| James M. Easton | Pennsylvania. |
| Herbert H. Emley | New Jersey. |
| Edward A. Ferbach | Germany. |
| Frank R. Fisk | Minnesota. |
| Henry J. Fisk | Connecticut. |
| Elmer E. Fleming. | Pennsylvania. |
| J. Glen Fling | Pennsylvania. |
| Oscar Gerdtzen | Chili, S. A. |
| Rudolph Gilgenberg | Germany. |
| Paul L. Gibson | New York. |
| Leon Goble | New Jersey. |
| Paul Guye | Switzerland. |
| Charles F. Hager | Pennsylvania. |
| Eugene C. Honeywell | Pennsylvania. |
| Mason P. Harvey | Maine. |
| Towsend H. Jacobs | Minnesota. |
| J. Stewart Jackson | New York. |
| F. H. Kendrick | Massachusetts. |
| Richard Knoblesdorff | Germany. |
| Paul Karrer | Switzerland. |
| Alfred H. Little | New York. |
| Oliver P. Lund | Pennsylvania. |
| Richard Longe | Germany. |
| Percival E. Loder, MD | Pennsylvania. |
| Roscoe Murphy | New Jersey. |
| A. How'd Macpherson | New Jersey. |
| John A. McClellan | Ohio. |
| Ferdinand E. Mueller | Germany. |
| E. M. S. McKee | Pennsylvania. |
| Rush J. McHenry | Pennsylvania. |
| Wilmer D. McKissick | Pennsylvania. |
| George C. Moore | Pennsylvania. |
| Samuel E. Marshall | Delaware. |
| Herman Reamer | Pennsylvania. |
| Delfin F. Restrepo | U. S. of Columbia |
| David S. Reed | Pennsylvania. |
| Anna B. Ramsay | Pennsylvania. |
| Albert Rentzing | Germany. |
| William F. Ravel. | France. |
| Marvin L. Rowe | Canada. |
| Alfred Steiger | Switzerland. |
| William S. Sullivan | Wisconsin. |
| George H. Swift | Vermont. |
| E. A. Shulenberger | Pennsylvania. |
| Edgar W. Smith | Maryland. |
| Michael Jos. Spahn | Germany. |
| Wilhelm Seltzer | Germany. |
| David P. Tait | Pennsylvania. |
| C. O. Tupper, A. B. | Canada. |
| Charles E. Walrad | New York. |
| Henry Weston | Pennsylvania. |
| Frank W. Williams | Massachusetts. |

Number of matriculates, one hundred and thirty-six. Graduates, sixty-nine.

### University of Tennessee—Dental College.

The seventh commencement was held Feb. 24th.

The following persons received the degree of D.D.S.:

| NAME. | RESIDENCE. |
|---|---|
| Samuel B. Anderson | Tennessee. |
| A. Y. Cartwright | South Carolina. |
| T. S. Cartwright | Kentucky. |
| Southall Dickson | Tennessee. |
| George W. Dodson | Tennessee. |
| James M. Glenn. | Tennessee. |
| Robert S. Griggs | Georgia. |
| H. D. Harper | North Carolina |
| Hardy B. Harrell | Georgia. |
| John F. Johnson | Mississippi. |
| John A. Lee | Tennessee. |
| Miles M. Puckett | Georgia. |
| M. E. Shelton | Missouri. |

Number of matriculates twenty-nine. Graduates, thirteen.

### Philadelphia Dental College.

The twenty-second annual commencement of this college was held Saturday, Feb. 28th.

| NAME. | RESIDENCE. |
|---|---|
| George H. Adair | New York. |
| A. S. Bailey | Massachusetts. |
| William Baylor | New York. |
| Noah S. Borneman. | Pennsylvania. |
| Edward H. Buckland. | Massachusetts. |
| C. J. Cameron | Pennsylvania. |
| Leopold A. Carter | Australia. |
| J. E. Cartland | North Carolina. |
| William G. Chase | Delaware. |
| Ernest Cohen | Germany. |
| George Eadie | Canada. |
| George F. English | New Jersey. |
| Mary E. Fetherolf | Pennsylvania. |
| H. A. Fynn | New York. |
| Isaac A. Goldsmith | South Carolina. |
| Harry P. Griffith | Pennsylvania. |
| William B. Griswold | Connecticut. |
| Henry J. Harwood | France. |
| Franklin L. Henry | Canada. |
| Burton E. Hoover | Pennsylvania. |
| Robert Sutcliffe Ivy | England. |
| A. W. Jamison | Pennsylvania. |
| Victor P. Janin | France. |
| Myron D. Jewell | New York. |
| Samuel H. Johns | Delaware. |
| R. Frank Jones | New York. |
| Augustus D. Josselyn | Maine. |
| Leonard F. Kellogg | Iowa. |

| NAME. | RESIDENCE. |
|---|---|
| Horace G. Lawson. | Canada. |
| Martin H. Lutz | Pennsylvania. |
| Edwin R. Magnus | Connecticut. |
| William T. Mahon | Pennsylvania. |
| W. R. Mail | Indiana. |
| Addison S. Melvin, jr. | Illinois. |
| Calvin R. Moulton | California. |
| Bruno Albert Nernst | Germany. |
| Robert H. Nones. | Pennsylvania. |
| Frank Delavan Norton | New York. |
| George D. O'Neil | Australia. |
| John F. O'Neil | Iowa. |
| Allison J. Parker | Massachusetts. |
| Myron F. Parmley | Iowa. |
| Charles M. Porter | Pennsylvania. |
| Edward W. Pratt | Connecticut. |
| James J. Ryan | Massachusetts. |
| Albert H. Schilt | Wisconsin. |
| Forbes C. R. Scott | France. |
| William S. Sherman | Rhode Island. |
| Frank M. Swain | Illinois. |
| Louis Teichmann | Germany. |
| Edgar W. Thompson | New York. |
| Charles P. Tuttle | New Jersey. |
| R. F. Verrinder, M.D. | California. |
| Hugh Walker | Canada. |
| Frank W. Wolf | Illinois. |
| Hiram M. Wolf | Illinois. |
| Morris Zeadler | Russia. |

Number of matriculates, one hundred and twenty-nine. Graduates, fifty-seven.

---

### Dental College of the University of Michigan.

The tenth regular Commencement of this College was held June 25th. The Degree of D.D.S. was conferred upon the following names:

| NAME. | RESIDENCE. |
|---|---|
| Lafayette Lyman Barber | Ohio. |
| William H. Bailey | Indiana. |
| Louis P. Bethel | Ohio. |
| Marian J. Coy | Ohio. |
| Homer A. Drake | Michigan. |
| John L. Gish | Indiana. |
| Elijah J. Galbraith Jr. | Ohio. |
| Fred S. Hadley | Wisconsin. |
| Elisie A. Hallock | Michigan. |
| George B. Haught | Ohio. |
| Daniel S. Husted | Ohio. |
| Charles W. Jones. | Wisconsin. |
| Leo Kratzsch | Wisconsin. |
| Herman C. Kuebler | Ohio. |

| FAME. | RESIDENCE. |
|---|---|
| Oscar B. Lundy | Michigan. |
| Clara W. McNaughton | Michigan. |
| Hervey C. Merrill | Germany. |
| Charles A. Murray | N. Brun. |
| Robert Nicol | Scotland. |
| William Morris Peck | Michigan. |
| Edward Fitz Randolph | Ohio. |
| Frank W. Ryan | N. Brun. |
| John Duncan Robertson | Michigan. |
| Willoughby B. Smith | Michigan. |
| May C. Smith | Illinois. |
| Isaac A. Stitt | Indiana. |
| Robert F. Taggert | Michigan. |
| Will H. Whistlar | Ohio. |

Number of Matriculates, eighty-two. Graduates, twenty-eight.

### University of Pennsylvania—Department of Dentistry.

The Annual Commencement of this Department was held on Friday, May 1st. The Degree of D.D.S., was conferred upon the following:

| NAME. | RESIDENCE. | NAME. | RESIDENCE. |
|---|---|---|---|
| William Y. Allen | Massachusetts. | L. Foster Jack, M.D. | Pennsylvania. |
| Edgar C. Baily | Georgia. | Victor S. Jones | Pennsylvania. |
| Edward R. Bellis | Indiana. | Frank C. Knapp | New York. |
| F. W. Bromley, M. D. | Wisconsin. | William R. Long | Pennsylvania. |
| Augustus Bruns | Germany. | Adelbert W. Marsh | New York. |
| Ditson P. Carter | Ohio. | Charles J. Mercer | Pennsylvania. |
| William S. Couto | Brazil. | George G. Milliken | Pennsylvania. |
| Ira B. Crissman | Illinois. | Charles T. Milliken | California. |
| Chas. Elmer Dayton | Connecticut. | Grafton Munroe | Maryland. |
| George W. C. Dick | South Carolina. | Edwin G. Parker | New York. |
| Edward B. Dickinson | Massachusetts. | Edwin Peardon | Wisconsin. |
| Robert Fulton Ehni | Ohio | Clarence F. Roett | Barbadoes W.I. |
| Geo. Ægidius Englert | New Jersey. | Jos. Roque | Cuba. |
| George W. Erskine | Pennsylvania. | George B. Ryan | Canada. |
| Oswald Fergus | Scotland. | John A. Schmidt | New York. |
| Frank W. Fisher | New York. | Louis Shaw | Pennsylvania. |
| Andreas Fridman | Sweden. | Chas. Elmer Smith | Iowa. |
| Clifford Gibbens | England. | W. Albert Smith | Colorado. |
| John F. Gibbons | England. | Claude A. Southwell | Wisconsin. |
| Louis Ginoyer | Brazil. | Ernest L. Southworth | California. |
| Thaddeus T. Hayward | Minnesota. | J. Walter Stapleton | Kentucky. |
| John C. Hertz | Pennsylvania. | R. Walter Starr | Pennsylvania. |
| Edward D. Howe | New Hampshire. | Henry M. Stine | Pennsylvania. |
| A. C. Hugenschmidt | France. | George H. Wells | Massachusetts. |

William L. Winner .... Pennsylvania.

The number of matriculates, one hundred and twelve. Graduates, forty-nine.

### Vanderbilt University—Dental Department.

The sixth annual commencement of this department was held Wednesday, Feb. 25th.

The honorary degree of D.D.S. was conferred on James S. Franklin, M.D., of Tennessee.

The following are the graduates:

| NAME. | RESIDENCE. | NAME. | RESIDENCE. |
|---|---|---|---|
| Charles F. Barham | Arkansas. | Jesse W. Shoemaker | Alabama. |
| Geo. C. Cooper, M. D. | England. | Geo. W. Slaughter | Alabama. |
| Jonathan A. Ellard | Alabama. | Lawrence A. Smith | Mississippi. |
| John W. Fambrough | Georgia. | Geo. W. Stokes | South Carolina. |
| Frank H. Field | Georgia. | John T. Taylor | Tennessee. |
| James A. Frazier | Alabama. | Tyra F. Tynes | Mississippi. |
| David R. Garrison | Texas. | Joseph W. Peete | Florida. |
| Fred W. Gradolph | Ohio. | Pinckney L. Weekley | South Carolina. |
| Edwin L. Hays | Kentucky. | Thomas C. West | Mississippi. |
| Wm. H. Hogshead | California. | Sheridan A. Williams | South Carolina. |
| Robert H. McNair | Mississippi. | Lucius D. Wright | Tennessee. |
| Wm. C. Naff | Tennessee. | John Wood, L.D.S., R.C S., Edinburg and Ireland, of Dumfries, Scotland. | |
| Clarence V. Rosser | Georgia. | | |

Number of matriculates for the session fifty-five. Graduates, twenty-six.

### New York College of Dentistry.

The nineteenth commencement of this institution was held on Monday, March 9th.

The following received the degree of D.D.S. :

| NAME. | RESIDENCE. |
|---|---|
| Fremont Allen | New York. |
| Lyndon C. Allen | New York. |
| Wm. F. Atkinson | New York. |
| Louis Arndt | New Jersey. |
| Enrique G. de la Beldad | Spain. |
| Ramon G. de la Beldad | Spain. |
| Lemuel P. Blair | Maine. |
| Max. R. Brinkman | Massachusetts. |
| Herbert W. F. Cady | New York. |
| Robert H. Cochran | Massachusetts. |
| Frank S. Crane | New Jersey. |
| Martin Degenhart | New York. |
| Arthur Dodge | Connecticut. |
| John Dunges | Germany. |
| Charles H. Eagleton | New York. |
| Thomas A. Fitzpatrick | New York. |
| Edward S. Fonda, M.D. | New York. |
| Frederick W. Gibbs | Connecticut. |
| Arthur F. Hawes | New Jersey. |
| Ignacia V. Herrera | Cuba. |
| George J. Harnung | New Jersey. |
| Joseph M. Henriques | West Indies. |
| Charles W. Howard. | New York. |
| Henry J. Hull | New York. |
| Lyman S. King | New York. |
| Daniel W. Klienhaus | New Jersey. |
| Leonard K. Knox | New York. |
| Allen S. McDougal | New York. |
| Joseph W. Moore | New York. |
| Wellslake D. Moore | New York. |
| Charles J. Mooney | New York. |
| Henry L. O'Brien | New York. |
| Frederick W. Pape | Germany. |
| Virgil F. Parker | New York. |
| Louis A. Queen | New Jersey. |
| Julius W. Rivinius | New York. |
| Livingston J. Roberts | New York. |
| John Roberts | Wales. |
| Joseph N. Shenstone | New York. |
| Samuel Simon | Massachusetts. |
| Karl C. Smith | New York. |
| Charles R. Smith | New Jersey. |
| Arthur L. Swift | New Jersey. |
| Louis M. Villalon | Porto Rico. |
| Johan A. Theo. Weber | Finland. |
| William M. Wyant | New York, |

Number of matriculates one hundred and sixty-five. Graduates, forty-six.

### University of Maryland—Dental Department.

The third annual commencement of this department was held on Tuesday, March 17th. The degree of D.D.S., was conferred upon the following :

| NAME. | RESIDENCE. |
|---|---|
| Madison A. Bailey | South Carolina. |
| E. Payson Beadles | Virginia. |
| H. Clinton Bradford | Virginia. |
| Claude D. Brown | Virginia. |
| John P. Carlisle | South Carolina. |
| Joseph W. Carter | Missouri. |
| Thomas M. Comegys | Tennessee, |
| Frank J. Cooke | Texas. |
| Will Edward Dorset | Virginia. |
| Joseph Fournier, jr. | New York. |
| Ferdinand Groshans | Maryland. |
| Charles W. Hebbel | Maryland. |
| John W. Helm | Maryland. |
| Charles E. Hill | Australia. |
| Ulysses S. Hougland | Indiana. |
| Clarence H. Howland | D. Columbia. |
| A. Hersey Howlett | Pennsylvania. |
| Peyton Hundley | Virginia. |
| Eli E. Josselyn, M.D. | N, Brunswick. |
| John S. Kloeber | Virginia. |
| Augustus Matthews | North Carolina. |
| Robert T. McQuown | Virginia. |
| William P. McQuown | Virginia. |
| W. McIntosh Norwood | South Carolina. |
| Will W. Parker | Minnesota. |
| Henry Clay Pitts | North Carolina. |
| Capers D. Perkins | Georgia. |
| James M. Ranson, jr. | West Virginia. |
| Brooks Rutledge | South Carolina. |
| Charles T. Schaer | Maryland. |
| Wm. Sherman Trapp | Pennsylvania. |
| Fred. A. Twitchell | Minnesota. |
| Albert Wangemann | Germany. |
| Floyd J. Welch | Virginia. |
| William F. Wegge | Wisconsin. |
| Frank Le Roy Wood | Maine. |

The number of matriculates for the term was seventy-four.

### Boston Dental College.

The Eighteenth Commencement of this College was held April 1st.

| NAME. | RESIDENCE. | NAME. | RESIDENCE. |
|---|---|---|---|
| Edwin Oscar Blanchard. | | Fred Harris Metcalf | Vermont. |
| Eugene M. Brown. | Massachusetts. | Fred Carville Merrill | Massachusetts. |
| Frank Herman Clock. | Massachusetts. | Josesph A. Moriarty | Massachusetts. |
| Herbert D. Currie | New Brunswick. | C. F. A. Pagenstecher. | West Indies. |
| Elmer Ellsworth Eddy. | Vermont. | James Earl Pease | Massachusetts. |
| Wilmer Stuart Elliott. | Massachusetts. | Charles B. Pierce | Massachusetts. |
| Edmund G. Flint | Massachusetts. | Freidol S. Putnam | New Hampshire. |
| Charles Aaron Fox. | Massachusetts. | William R. Sawyer | Massachusetts. |
| George E. Hathorne | Main. | Cyrus Tyler Sherman. | Massachusetts. |
| Charles E. Helah | Main. | William H. Spencer | New York. |
| George H. Hoadley. | Vermont. | Oscar Herbert Stevens. | Massachusetts. |
| Harvey Martelle Kirk | Ohio. | William C. Stoddard | Rhode Island. |
| Frank Winslow Kyes. | New Hampshire. | Leslie Mitchell Swett. | Massachusetts. |
| Charles A. Leslie | Massachusetts. | | |

Graduates, twenty-seven.

### The University of Iowa--Dental Department.

The third annual commencement of this department was held on Monday evening, March 2d. The degree of D.D.S. was conferred upon the following:

| NAME. | RESIDENCE. | NAME. | RESIDENCE. |
|---|---|---|---|
| Emory L. Brooks | Iowa. | J. C. Mitten | Iowa. |
| Henry Clemens | Iowa. | H. M. McAlister | Iowa. |
| H. M. Dalzell | Iowa. | J. A. Ross | Iowa. |
| L. K. Fullerton | Iowa. | F. H. Rule | Iowa. |
| G. E. Fisher | Iowa. | H. H. Smith | Iowa. |
| H. A. Harlan | Iowa. | J. L. Small | Iowa. |
| John P. Hunt | Iowa. | C. G. Thomas | Iowa. |
| J. C. Holland, M.D | Iowa. | S. R. Wagoner | Montana. |

The number of matriculates thirty-five. Graduates, sixteen.

### Indiana Dental College.

The sixth commencement was held March 4th.

The following-named persons graduated:

| NAME. | RESIDENCE. | NAME. | RESIDENCE. |
|---|---|---|---|
| William C. Archer | Indiana. | Frank H. Horner | Pennsylvania. |
| William H. Bucher | Indiana. | Marshall M. Keep | Pennsylvania. |
| Frank Dowd | New York. | B. G. Miller | Michigan. |
| John E. Davis | Indiana. | James W. Pfall | Indiana. |
| Williem R. Dunn | Indiana. | J. Monticello Sprinkle | Indiana. |
| Fred. H. Emmerling | Wisconsin. | George W. Tainter | Missouri. |
| Gust. Weinmann | Pennsylvania. | | |

Matriculates during the term, twenty-seven. Graduates, thirteen.

### Chicago College of Dental Surgery.

The third annual commencement of this College was held on Friday, March 27th. The Degree of D.D.S. was conferred upon the following:

| NAME. | RESIDENCE. | NAME. | RESIDENCE. |
|---|---|---|---|
| H. Austin Armitage, M.D. | England. | William J. Johnson, M.D. | Illinois. |
| Harry Leon Barnum, M.D. | Wisconsin | Edmund Lambert | Illinois. |
| Edward Everett Cady | Illinois. | Asa Holt Lane | Illinois. |
| Warren Cary, M.D. | Illinois. | Charles William Lewis | Illinois. |
| Jesse Austin Dunn | Illinois. | Archibald S. McCandless | Illinois. |
| Astor Gerard Gray | Illinois. | Joseph Donahey Moody | Illinois. |
| Rudolph Theodore Hasselriis | Denmark. | Amos Jedd Nichols | Illinois. |
| Joseph Hickey | Dakota. | Charles Putnam Pruyn | Illinois. |
| John Edward Hinkins | Illinois. | Joseph J. Reed | Illinois. |
| A. Melville Hudson | Canada. | Charles Henry Wachter | Maryland. |
| Charles N. Johnson, L.D.S. | Ontario. | George W. Whitefield | Illinois. |

The number of matriculates, sixty-two. Graduates, twenty-two.

### Minnesota College Hospital—Dental Department.

The annual Commencement of this Department was held on the evening of February 27th. · The Degree of D.D.S. was conferred upon the following:

| NAME. | RESIDENCE. | NAME. | RESIDENCE. |
|---|---|---|---|
| John H. Spalding | Minnesota. | Charles L. Opsal | Minnesota. |
| John H. Dwight | Minnesota. | | |

Graduates, three.

### Missouri Dental College.

The nineteenth commencement was held March 6th.

| NAME. | RESIDENCE. | NAME. | RESIDENCE. |
|---|---|---|---|
| J. H. Bland | Missouri. | T. A. Williamson | Minnesota. |
| G. V. Collins | Illinois. | H. C. Miller | Missouri. |
| C. B. Helm | Illinois. | S. T. Neill | Missouri. |
| M. D. La Croix | Illinois. | W. H. Wright | Missouri. |

Number of Matriculates, nineteen. Graduatues, eight.

### Kansas City Dental College.

The annual commencement of this college was held on Tuesday, March 17th. The degree of D.D.S. was conferred on the following:

| NAME. | RESIDENCE. | NAME. | RESIDENCE. |
|---|---|---|---|
| W. M. Dunning | Kansas. | John E. Crozier | Missouri. |
| H. I. Parr | Kansas. | J. W. Buchanan | Missouri. |

Matriculates, eight. Graduates, four.

### University of California—Dental Department.

The third annual commencement exercises of this College were held on Tuesday evening, November 11, 1884.

The degree of D.D.S., was conferred on the following named persons:

| NAME. | RESIDENCE. | NAME. | RESIDENCE. |
|---|---|---|---|
| George Washington Cool | California. | Gustavus Carl Rietzke | Germany. |
| John Michael Dunn | California. | William Henry Simmons | California. |
| Archibald Duncan Gleaves | California. | Elmer Joseph Weldon | California |
| Robert Nicol | Scotland. | | |

Graduates, seven.

### National University—Dental Department.

The first annual commencement was held in Washington, D.C., on May 22. The degree of D.D.S. was conferred on the following persons:

| NAME. | RESIDENCE. | NAME. | RESIDENCE. |
|---|---|---|---|
| Edwin Howard | Dist. of Columbia. | L. A. Brown | West Virginia. |
| C. M. Kennedy | Dist. of Columbia. | G. W. Egleston | New York. |

